# Patient-Reported Outcome Measures for Assessing Dual-Task Performance in Daily Life: A Review of Current Instruments, Use, and Measurement Properties

**DOI:** 10.3390/ijerph192215029

**Published:** 2022-11-15

**Authors:** Zuhal Abasıyanık, Renee Veldkamp, Amber Fostier, Carolien Van Goubergen, Alon Kalron, Peter Feys

**Affiliations:** 1 REVAL Rehabilitation Research Center, Faculty of Rehabilitation Sciences, Hasselt University, Martelarenlaan 42, Agoralaan 1, 3500 Hasselt, Belgium; 2 Graduate School of Health Sciences, Dokuz Eylül University, Izmir 35220, Turkey; 3 Department of Physiotherapy and Rehabilitation, Faculty of Health Sciences, Izmir Katip Celebi University, Izmir 35620, Turkey; 4 Universitair MS Centrum, 3500 Hasselt, Belgium; 5 Department of Physical Therapy, Sackler Faculty of Medicine, School of Health Professions, Tel-Aviv University, Tel-Aviv 69978, Israel

**Keywords:** dual-task, cognitive-motor interference, patient-reported outcome measures, psychometric properties, validity, reliability

## Abstract

The patient perspective of dual-task (DT) impairment in real life is unclear. This review aimed (i) to identify patient-reported outcome measures (PROMs) on DT and evaluate their measurement properties and (ii) to investigate the usage of PROMs for the evaluation of DT difficulties. A systematic literature search was conducted using PubMed and Web of Science from inception to March 2022. Methodological quality was evaluated using the COSMIN checklist. Six studies examined the measurement properties of DT PROMs. Nine studies used DT PROMs as the outcome measure. Five PROMs were identified, including the Divided Attention Questionnaire (DAQ), Dual-Task-Impact on Daily-life Activities Questionnaire (DIDA-Q), a Questionnaire by Cock et al. (QOC), Dual-Tasking Questionnaire (DTQ), and Dual-Task Screening-List (DTSL). Fourteen measurement properties were documented: five (35.7%) rated quality as “sufficient”, six (42.8%) “insufficient”, and three (21.4%) “indeterminate”. The quality of evidence for each measurement property ranged from very low to high. While DT performance is investigated in many populations, the use of PROMs is still limited, although five instruments are available. Currently, due to insufficient data, it is not possible to recommend a specific DT PROM in a specific population. An exception is DIDA-Q, which has the highest quality of measurement properties in people with multiple sclerosis.

## 1. Introduction

Daily life activities generally require performing a secondary cognitive or motor task [i.e., dual-task (DT)], such as walking while talking or eating while listening. Recent studies showed that DT walking speed measured in the laboratory was lower than during single walking but similar to the most used walking speed in daily life [1,2]. Therefore, the measurement of DT walking performance has received much attention as it is thought to better reflect everyday life conditions and thereby provide an improved outcome for ecological validity.

An increasing amount of research has examined DT performance, using different cognitive tasks and various walking and balance tasks in many populations, predominantly in neurological diseases (e.g., Parkinson’s disease, multiple sclerosis, stroke, mild cognitive impairment, dementia, traumatic brain injury), elderly, children, and healthy adults [3,4,5,6,7]. Studies generally show that when the motor and cognitive tasks are combined, it can lead to worse performance in one or both tasks, particularly in the elderly and people with neurological diseases [3,8,9]. The association between a decline in performance and aspects of daily life, such as an elevated risk of falling and a lower quality of life, led to increased interest in intervention strategies [6]. Furthermore, several previous studies examined the psychometric (also called measurement) properties of DT assessments to provide accurate measurement, with some reliable and valid outcome measures identified for use in research and clinical settings [10,11,12].

However, besides the objective measurement procedures of DT walking collected in highly controlled lab settings, there is limited knowledge about perceived dual-task difficulties in daily life. Everyday life is typically different from traditional lab conditions, with varying and unpredictable distractors. In addition to walking and thinking, a wide variety of different motor and cognitive DT activities are required in daily life. Thus, the generalizability of DT lab outcomes to be transferred to real-life situations may be questioned and is poorly understood. Patient-reported outcome measures (PROMs) measuring different daily living activities are likely to capture a wider dimension than only a standardized DT walking test. Therefore, PROMs focusing on DT performances are essential [13].

To the best of our knowledge, there is no systematic review that has summarized the measurement properties (reliability, validity, and responsiveness) of PROMs for DT walking difficulties. This information is critical for research and clinical work in various populations. Therefore, the purpose of our systematic review is to explore: (i) the measurement properties, methodological quality, and descriptive characteristics of PROMs specific to DT activities and (ii) the use of PROMs for evaluation (or screening) of DT difficulties.

## 2. Materials and Methods

This systematic review was performed according to the Consensus-based Standards for the Selection of Health Measurement Instruments (COSMIN) methodology for systematic reviews of measurement properties (2018) for systematic reviews [14]. It has been provided to perform an updated, appropriate methodology for a systematic psychometric review. Therefore, it allows the selection of the instruments for research or clinical practice and identifies gaps in knowledge on the quality of measurement properties. This review was reported following the Preferred Reporting Items for Systematic Reviews and Meta-Analyses (PRISMA) guidelines [15]. The protocol was registered at the International Prospective Register of Systematic Reviews (PROSPERO Reference: CRD42022325230).

### 2.1. Eligibility Criteria

The inclusion criteria for study selection were: (1) developed patient-reported outcome measures (PROMs) to evaluate perceived DT difficulties in daily life and/or reporting at least one measurement property according to the COSMIN terminology and definitions or using PROMs as outcome measures or screening method (2) English language; (3) Full-text is available.

Conference proceedings, editorials, (systematic) reviews, meta-analyses, practice guidelines, letters, and animal studies were excluded from the study.

### 2.2. Search Strategy

A systematic search was performed using the MEDLINE and Web of Science databases on 23 March 2022 without date restrictions. The Medical Subject Headings terms (MeSH-terms) and keywords were selected based on their relevance to the research question. By adding the Boolean operators AND and OR accordingly, the following complete search strategy was constructed: questionnaire AND dual task OR “cognitive motor interference” OR “divided attention”. In addition, reference lists of all included studies were thoroughly examined to detect any other potentially eligible papers for inclusion.

### 2.3. Study Selection

Two reviewers (A.F. and C.V.G.) independently screened the articles by title and abstract. The reviewers extracted all potentially eligible articles from the title and abstract review and retrieved the full text. In cases where the full text was unavailable, a request was sent to the corresponding author. The two reviewers then discussed the findings and reached a consensus on the final articles to be included for further analysis. In case of inconsistency and/or disagreement, a third reviewer was consulted (Z.A.).

### 2.4. Data Extraction

The following data were extracted from each included study: basic characteristics of the study (authors, year of publication, etc.), details of the study design (sample size, aims, type of the study, etc.), participant characteristics (population used for the validation process, age, gender, etc.), details of the PROM (name, number of items, subscales, response options, scoring methodology, and range of scores), and measurement properties according to the COSMIN guideline. The main results of the included studies were also retrieved.

Additionally, for each study included in the final analysis, we evaluated the content validity, the internal structure (including structural validity, internal consistency, and cross-cultural validity), reliability, measurement error, criterion validity, hypothesis testing for construct validity, and responsiveness when available. Definitions of these measurement properties and taxonomy can be found in the COSMIN manual for systematic reviews of PROMs [14].

### 2.5. Quality Assessment

The methodological quality of each eligible study was independently ranked by two researchers (A.F. and C.V.G.) utilizing the COSMIN Risk of Bias checklist. The quality of the methodology employed in each study to determine the measurement property was then independently graded on a four-point scale: very good, adequate, doubtful, and inadequate. The lowest rating of any standard within a box was used as the rating for that measurement property (worst score counts principle) [16].

The results from each study on a measurement property were assigned a quality rating as sufficient (+), insufficient (−), or indeterminate (?) [14].

### 2.6. Summary and Grading of the Quality of Evidence

This section refers to rating the quality of the PROM as a whole. PROMs were qualitatively summarized and assigned a four-point quality rating. A modified Grading of Recommendations Assessment, Development and Evaluation (GRADE) approach (omitting publication bias) was used to assign evidence quality as high, moderate, low, or very low [14].

## 3. Results

**Figure 1:**
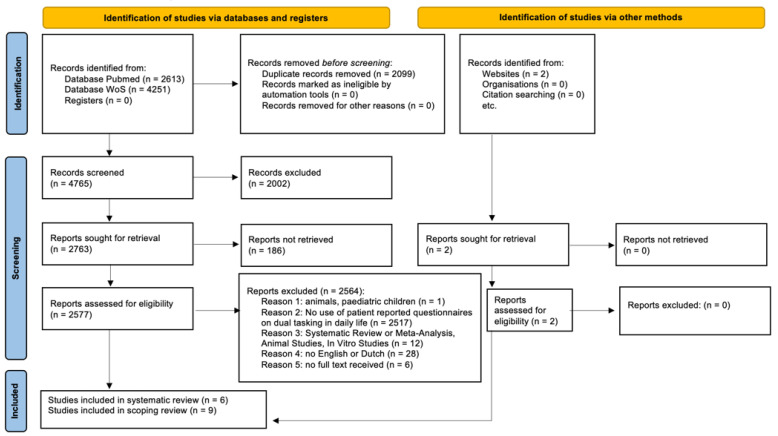
Flowchart of the study.

A total of 6864 records were identified, of which 4765 articles were screened on title and abstract following the removal of duplicates. Of those screened, 2577 full-text publications were assessed for eligibility, and six articles providing measurement properties were included in the outcome measure evaluation in the systematic review. Additionally, nine studies using PROMs as outcome measures or the screening method were included for documenting current use. Figure 1 demonstrates the PRISMA flow diagram. No meta-analysis was performed due to the heterogeneity of the outcomes and study designs of included studies.

### 3.1. Description of PROMs Assessing Dual-Task Difficulties

Five PROMs were identified: Divided Attention Questionnaire (DAQ), Dual-Task-Impact on Daily-life Activities Questionnaire (DIDA-Q), Questionnaire by Cock et al. [17] (QOC), Dual-Tasking Questionnaire (DTQ), and Dual-Task Screening-List (DTSL). The DTQ, DIDA-Q, QOC, and DTSL have been developed for persons with neurological diseases (traumatic brain injury and stroke, multiple sclerosis, acquired brain injury, and Parkinson’s disease, respectively). The DAQ has been developed for older and young adults. The DIDA-Q was developed in Italian, and the DTSL in Dutch, but translated English versions have since been published [18,19]. While DTQ, DTSL, and QOC were developed and immediately used as outcome measures in planned research (intervention and observational studies), the authors mainly introduced a new instrument for DAQ and DIDA-Q. Table 1 provides a full explanation of the PROMs.

### 3.2. Methodological Quality of the Included Studies on Measurement Properties

The methodological quality of included studies is presented in Table 2. A total of 22 measurement properties were evaluated in the included studies. Six measurement properties (27.3%) were rated as having “very good”, five (22.7%) “adequate”, eight (36.4%) “doubtful”, and three (13.6%) “inadequate” methodology quality.

The quality of the PROM development process for the DAQ (in young and older adults), DIDA-Q (in persons with multiple sclerosis), and QOC (in persons with acquired brain injury) is presented in Table 3 [17,18,23]. It was unclear whether the study involving the QOC was a PROM development study. Nevertheless, we rated it because it provided information on PROM development [17]. Although DIDA-Q is generally scored as “very good” in PROM design items and performed in a pilot test [18], all three questionnaires are rated as “doubtful” in total due to the “worst score counts” principle by COSMIN.

### 3.3. Validity Measures of PROMs Assessing DT Difficulties

Table 4 presents the overall evidence for each measurement property against the COSMIN GRADE Assessment. Table 5 details the measurement properties separately for each included study. Five studies investigated the validity of three PROMs (DAQ, DTQ, and DIDA-Q).

The structural validity of DIDA-Q was rated “sufficient” (with moderate quality of evidence) in people with multiple sclerosis (MS). In contrast, the structural validity of the DAQ and QOC was rated as “insufficient” (with low and very low quality of evidence) in adults and persons with brain injury, respectively.

Four studies reported correlations between DT PROMs and other outcome measures (i.e., hypothesis testing) for the construct validity of DAQ (in young and older adults), DTQ (in older adults), DIDA-Q (in MS), and QOC (in acquired brain injury) [17,18,20,21]. The overall rating was “insufficient” for DAQ, “indeterminate” for DTQ and QOC, and “sufficient” for DIDA-Q. The quality of evidence was rated very low (DAQ, DTQ, and QOC) to moderate (DIDA-Q). The comparison between subgroups (i.e., known groups or discriminative validity) was documented only for DIDA-Q, and significant differences were noted for different disability levels in persons with MS [18].

Cross-cultural adaptation was only performed by Sertel et al. and Amini et al. [20,21]. The DTQ has been translated from English to Turkish and adapted for older adults, and DAQ has been translated into Persian. Cross-cultural validity (differential item functioning between languages) has not been investigated in any study.

### 3.4. Reliability Measures of PROMs Assessing DT Difficulties

Five studies reported the internal consistency of three PROMs:DAQ, DTQ, and DIDA-Q. The overall rating was “insufficient” with moderate quality of evidence for DAQ, “insufficient” with very low quality for DTQ, and “sufficient” with high quality for DIDA-Q. Cronbach’s alpha scores are provided in Table 5. Cronbach’s alpha was ≥0.70 (statistically acceptable value) for individuals with MS on the DIDA-Q and for young and older adults on the DAQ.

Test-retest reliability was reported in five studies for DAQ, DTQ, DIDA-Q, and QOC. The overall rating was “insufficient” with moderate quality of evidence for DAQ, “sufficient” with very low quality for DTQ, “sufficient” with moderate quality for DIDA-Q, and “indeterminate” with very low quality for QOC. For QOC, Cohen’s coefficient was calculated instead of the intraclass correlation coefficient (ICC) in six persons. ICC scores for each PROM are provided in Table 5.

### 3.5. Characteristics of Studies Using PROMs on DT as an Outcome Measure or for Participant Screening

Table 6 details the nine studies that used PROMs as an outcome measure or for screening purposes. The most commonly measured population was MS (*n* = 5), followed by Parkinson’s disease (*n* = 1), mild cognitive impairment (*n* = 1), acquired brain injury (*n* = 1), and spinal cord injury (*n* = 1). DAQ and DTQ were used as outcome measures, while DTSL was used for the inclusion criteria of an intervention study. In four studies, DTQ scores were compared between persons with MS and healthy controls, finding significant differences between groups that support the discriminative validity of the DTQ in MS [12,24,25,26]. Three studies used PROMs (DTQ, DAQ, DTSL) as an experimental outcome measure in intervention studies. The DTQ improved following a cognitive-motor DT exercise training program in individuals with acute brain injury but not in persons with MS [27,28]. Improved scores on the DAQ were found following a training program based on divided attention (cognitive-cognitive) in persons with mild cognitive impairment. [29]. DTSL was used as an inclusion criterion in four studies (*n* = 3 in MS, *n* = 1 in Parkinson’s disease) [12,24,28,30], two of which were intervention studies [28,30].

## 4. Discussion

This review provides an overview of the use of PROMs assessing DT difficulties and synthesizes the current evidence from six studies that aimed to evaluate the measurement properties of these PROMs. To the best of our knowledge, this is the first review that investigated PROMs assessing perceived DT difficulties. A total of 14 measurement properties were documented, with 5 (35.7%) rated as “sufficient”, 6 (42.8%) “insufficient”, and 3 (21.4%) “indeterminate” quality. Five PROMs were identified that were developed for different populations and used as outcome measures. No measurement properties of the DTSL were examined, and the quality of evidence of DTQ, DAQ, and QOC for reliability and validity was generally rated very low and low. The DIDA-Q obtained the highest number of positive criteria for measurement properties, although solely in MS.

The DIDA-Q, which consists of the most items and includes subscales of cognition, upper extremity, and balance-mobility, has been investigated only in persons with MS from Italy [18]. The DIDA-Q presents sufficient internal consistency, reliability, structural validity, and construct validity. Still, the PROM development was rated “doubtful” in our review. Furthermore, the measurement error and cross-cultural validity of DIDA-Q have yet to be investigated. Given the moderate to high-quality evidence for many measurement properties, we recommended its use in the MS population. Nevertheless, we encourage future studies to continue investigating its measurement properties in other populations and languages.

According to our findings, all studies using PROMs for perceived DT difficulties as an outcome measure were in people with neurological diseases highlighting its necessity in these populations. Only two (DIDA-Q and DAQ) PROMs have provided detailed information on the development procedures. The low methodological quality scores of the other PROMs are likely because they did not conduct the PROM development processes systematically. Therefore, we recommend that future PROM development and cross-cultural adaptation studies on perceived DT difficulties follow COSMIN tools.

Although the purpose of all five DT PROMs is to evaluate DT difficulties, instrument properties differ among PROMs. The DTSL was designed as a checklist (yes/no choice); others were Likert-type scales to determine the difficulty level. There is no study on the measurement properties of DTSL. However, it was utilized to show the presence of DT impairment as an inclusion criterion in some intervention and cross-sectional studies in persons with MS and Parkinson’s disease [12,24,28,30]. Although we think that the use of a checklist for DT training and assessment studies is relevant, there is a need for a study to explore discriminative and other measurement properties.

Cross-cultural adaptation studies are essential, allowing researchers and health professionals in different societies to acquire comparable data for DT difficulties. We observed that only DTQ and DAQ had been culturally adapted for use in other languages (Turkish and Persian) [20,21]. We recommend performing cross-cultural adaptation studies with rigorous methodologies.

Identified PROMs generally showed deficiencies regarding responsiveness, measurement error, cross-cultural validity, and discriminative validity. It is important to emphasize these flaws for future studies in clinical and research contexts. While the criterion validity is not applicable, it is essential to determine the relevance to the lab-based DT performance tests commonly used to detect DT impairment so far. Only one study has examined the relationship between the Timed-Up and Go test with a cognitive task and the DTQ, and authors find small significant correlations between perceived DT impairment and lab-based DT test in older adults [20].

### Methodological Considerations

A major strength of this review is the use of the updated version COSMIN methodology for systematic reviews of measurement properties for systematic reviews.

No meta-analysis was performed due to the heterogeneity of the outcomes and study designs of included studies. It is noted that only a limited number of questionnaires were found. However, this is likely as DT assessment and treatment is a relatively new domain of investigation.

## 5. Conclusions

This review highlights the importance of understanding the quality of PROM development and measurement properties of PROMs for proper use and interpretation in a particular population. Based on the evidence from this review, we recommend utilizing the DIDA-Q to assess perceived DT difficulties in persons with MS. The measurement properties of the DTSL were not investigated, and the quality of evidence of the DTQ, DAQ, and QOC was usually rated as very low and low. The responsiveness, measurement error, and cross-cultural validity of the identified PROMs have yet to be studied. We acknowledge that further studies focusing on measurement errors, cross-cultural validity, and comparison with lab-based DT walking assessments are warranted.

## Figures and Tables

**Table 1:** Characteristics of PROMs assessing dual-task difficulties.

PROMs	Population Developed (Language)	Adapted Population(s) (Language)	Development Process	Purpose of Development	Instrument Properties	Response Options	Scoring
Dual-task Questionnaire (DTQ)	Traumatic brain injury and stroke (English)	Older adults (Turkish)	No information	Using as an outcome measure for the intervention study	10 items, no subscales	5-point Likert-type scale [0 (never) to 4 (very often)]	Range: 0–40 Higher scores reflect higher dual-task impairment
Dual-task Screening List (DTSL)	Parkinson’s Disease (Originally in Dutch, provided in English)	-	No information	Using a screening list for the intervention study	13 items, no subscales	Checklist [0 (no) to 1 (yes)]	Range: 0–12 Higher scores reflect higher dual-task difficulties
Dual Task Impact on Daily Living Activities Questionnaire (DIDA-Q)	Persons with Multiple Sclerosis (Originally in Italian, provided also in English)	-	Literature review, Expert panel discussions	Development of a new instrument	19 items, three subscales (cognition, upper-extremity, balance-mobility)	5-point Likert-type scale [0 (not difficult) to 4 (extremely difficult)]	Range: 0–76 Higher scores reflect higher dual-task difficulties
Divided Attention Questionnaire (DAQ)	Older and young adults (English)	University students (Persian)	Discussing with participants and colleagues	Development of a new instrument	15 items	Difficulty: 5-point Likert-type scale [1 (very easy) to 5 (very hard)] Change: 3-point Likert-type scale [1 (easier), 2 (no change), 3 (harder)] Frequency: 3-point Likert-type scale [1 (0 per week), 2 (1–6 times per week), 3 (>6 times per week)]	Range: Difficulty scale: 16–80 Change scale: 16–48 Frequency scale: 16–48 Higher scores reflect higher difficulty, higher changes, and higher frequency
Questionnaire by Cock et al. (QOC) [17]	Acquired brain injury (English)		Discussing with participants and colleagues	Using as an outcome measure to compare the perceptions of staff and patients’ dual-task difficulties	8 items, no subscales	5-point Likert-type scale [1 (definitely) to 4 (definitely not)]	Range: 0–32 Higher scores reflect higher dual-task difficulties

**Table 2:** Quality of studies on measurement properties.

	DTQ	DAQ			DIDA-Q	OQC
	Sertell et al. (2021) [20]	Amini et al. (2016) [21]	Salthouse and Siedlecki (2005) [22]	Tun and Wingfield (1995) [23]	Pedullà et al. (2020) [18]	Cock et al. (2003) [17]
Structural Validity			D		A	D
Internal Consistency	V	V	V	V	V	
Cross-cultural Validity and Measurement Invariance						
Reliability	A		D	D	A	D
Measurement Error						
Criterion Validity	N/A	N/A	N/A	N/A	N/A	N/A
Construct Validity–Convergent validity	A	D	I	I	V	I
Construct Validity–Discriminative or known-group validity		N/A		D	A	D
Responsiveness						

Scores for methodological quality using the COSMIN Risk of Bias Checklist; available options are very good (V), adequate (A), doubtful (D), inadequate (I), or Not applicable (N/A); Empty cells indicate that the measurement property was not evaluated in this study; Abbreviations: DTQ = Dual Tasking Questionnaire; DAQ = Divided Attention Questionnaire; DIDA-Q = Dual Task Impact on Daily Living Activities Questionnaire; QOC = Questionnaire by Cock et al [17].

**Table 3:** Quality of PROM development.

			DAQ	DIDA-Q	QOC
PROM Design	General design requirements	Is a clear description provided of the construct to be measured?	V	V	V
		Is the origin of the construct clear: was a theory, conceptual framework, or disease model used, or was a clear rationale provided to define the construct to be measured?	V	V	V
		Is a clear description provided of the target population for which the PROM was developed?	V	V	V
		Is a clear description provided of the context of use?	D	V	D
		Was the PROM development study performed in a sample representing the target population for which the PROM was developed?	V	V	V
	Concept elicitation (relevance and comprehensiveness)	Was an appropriate qualitative data collection method used to identify relevant items for a new PROM?	V	V	D
		Were skilled group moderators/interviewers used?	D	N	N
		Were the group meetings or interviews based on an appropriate topic or interview guide?	N	N	N
		Were the group meetings or interviews recorded and transcribed verbatim?	N	N	N
		Was an appropriate approach used to analyze the data?	V	V	D
		Was at least part of the data coded independently?	D	N	D
		Was data collection continued until saturation was reached?	A	V	D
		For quantitative studies: was the sample size appropriate?	V	V	V
		Total quality of the PROM design	D	V	D
Cognitive interview (CI) study	General design requirements	Was the cognitive interview study or other pilot test performed in a sample representing the target population?		V	
		Comprehensibility		D	
		Comprehensiveness			
		Total CI score	D	D	D
Total quality of the PROM development study			D	D	D

Available options are V = very good; A = adequate; D = doubtful; I = inadequate; N = not applicable; Empty cells indicate that a related item was not performed; Abbreviations: PROM = patient-reported outcome measure; DAQ = Divided Attention Questionnaire; DIDA-Q = Dual Task Impact on Daily Living Activities Questionnaire; QOC = Questionnaire by Cock et al [17].

**Table 4:** Quality of the evidence for psychometric properties of the PROMs.

	DTQ		DTSL		DIDA-Q		DAQ		QOC	
	Overall Rating (+/−/?)	Quality of Evidence (High, Moderate, Low, Very Low)	Overall Rating (+/−/?)	Quality of Evidence (High, Moderate, Low, Very Low)	Overall Rating (+/−/?)	Quality of Evidence (High, Moderate, Low, Very Low)	Overall Rating (+/−/?)	Quality of Evidence (High, Moderate, Low, Very Low)	Overall Rating (+/−/?)	Quality of Evidence (High, Moderate, Low, Very Low)
Structural Validity					+	moderate	−	low	−	very low
Internal Consistency	−	low			+	high	−	moderate		
Reliability	+	very low			+	moderate	−	moderate	?	very low
Construct validity	?	very low			+	moderate	−	very low	?	very low

Available options are: + = sufficient, − = insufficient, ? = indeterminate; Empty cells indicate that related item was not performed; Abbreviations: PROM = patient-reported outcome measure; DTQ = Dual-task Questionnaire, DTSL = Dual-task Screening List, DIDA-Q = Dual Task Impact on Daily Living Activities Questionnaire; DAQ = Divided Attention Questionnaire; QOC = Questionnaire by Cock et al [17].

**Table 5:** Results of studies that assessed psychometric properties of PROMS.

Authors	PROM (Language)	Population	Mean Age	Results of Psychometric Properties					
				Reliability			Validity		
				Internal Consistency (Cronbach’s Alpha)	Test-Retest Reliability	Measurement Error	Construct Validity (Hypothesis Testing)	Construct Validity (Known-Groups, Discriminative Validity)	Structural Validity
Amini et al. (2016) [21]	DAQ (NR)	University students, *n* = 200	N/A	DAQ total = 0.67	NDA	NDA	DAQ and BIS-11: r = 0.41 DAQ and EMIS: r = −0.02 Priori hypotheses are not provided.	NDA	NDA
Cock et al. (2003) [17]	QOC (EN)	ABI, *n* = 50 Staff, *n* = 50	50.18 ± 16.47	NDA	NDA for ICC (Cohen’s k-coefficient = 0.6)	NDA	NDA	NDA	Exploratory factor analysis (2 factors)
Pedullà et al. (2020) [18]	DIDA-Q (IT)	MS, *n* = 230	52.8 ± 11.7	DIDA-Q total = 0.95 DIDA-Q M = 0.93 DIDA-Q = 0.90 DIDA-Q U = 0.90	DIDA-Q total: ICC = 0.95 DIDA-Q M: ICC = 0.76 DIDA-Q C: ICC = 0.76 DIDA-Q U: ICC = 0.81	NDA	DIDA-Q total and DAQ: r = 0.54 DIDA-Q and FIM: r = −0.55 DIDA-Q and SDMT: r = −0.36 DIDA-Q and ABILHAND: r = −0.61 DIDA-Q and MFIS: r = 0.67 70% of the hypotheses were confirmed.	Mild and moderate disability: DIDA-Q total: *p* < 0.0001 DIDA-Q M: *p* < 0.0001 DIDA-Q C: *p* = 0.0006 DIDA-Q U: *p* = 0.0002	Exploratory factor analysis (3 factors)
Salthouse and Siedlecki (2005) [22]	DAQ (EN)	Adults, *n* = 441	18–91	DAQ Difficulty scale = 0.87 DAQ Change scale = 0.81 DAQ Frequency scale = 0.79	Difficulty scale: r = 0.87 Change scale: r = 0.73 Frequency scale: r = 0.58	NDA	No relation was found between the DAQ and divided attention measures (range of r = 0.03 to 0.021) No confirmation of construct validity of DAQ.		Confirmatory factorial analysis and exploratory factor analysis (3 factors)
Sertell et al. (2021) [20]	DTQ (TR)	Older adults, *n* = 118	70.57 ± 5.83	DTQ = 0.695	ICC = 0.991	NDA	DTQ and TUG motor score: r = 0.350 DTQ and TUG cognitive score: r = 0.272 DTQ and Tinnetti gait score: r = −0.329 DTQ and Tinetti total score: r = –0.425, DTQ and Tinnetti Balance score: r = −0.444 Priori hypotheses are not provided.	NDA	NDA
Tun and Wingfield (1995) [23]	DAQ (EN)	Young, *n* = 83 Young-old, *n* = 114 Old, *n* = 104 Old-old, *n* = 27	19.3 ± 1.7 66.1 ± 3.1 75.9 ± 2.7 83.3 ± 5.3	DAQ Difficulty scale = 0.88 DAQ Change scale = 0.89 DAQ Frequency scale = 0.70	Difficulty scale: r = 0.63 Change scale: r = 0.44 Frequency scale: r = 0.52	NDA	NDA	NDA	Exploratory factor analysis for DAQ difficulty scale (3 factors)

Abbreviations: PROM = patient-reported outcome measure; DAQ = Divided Attention Questionnaire; DTQ = Dual Tasking Questionnaire; DIDA-Q = Dual Task Impact on Daily Living Activities Questionnaire; QOC Questionnaire by Cock et al [17]; TR = Turkish; EN = English; IT = Italian; NR = Not reported; MS = multiple sclerosis; ABI = acquired brain injury; NDA = no data available; BIS = Barratt Impulsiveness Questionnaire; EMIS = Eysenck and Murray Impulsivity Scale; r = Spearman or Pearson correlation coefficients; ICC = Intraclass Correlation Coefficient; DIDA-Q M = DIDA-Q subscale balance and mobility; DIDA-Q C = DIDA-Q subscale cognition; DIDA-Q U = DIDA-Q subscale upper limb abilities; FIM = Functional Independence Measure; SDMT = Symbol Digit Modalities Test; MFIS = Modified Fatigue Impact Scale.

**Table 6:** Results of studies that used PROMs as an outcome measure or screening method.

Authors	PRO	Design	Population	Mean Age	Results
Butchard-MacDonald et al. (2018) [25]	DTQ	NRCT	MS-RR, *n* = 34 HC, *n* = 34	43.1 ± 9.9 42.6 ± 10.1	DTQ was used as an outcome measure. Persons with MS-RR showed significantly higher scores on DTQ compared to HC (*p* < 0.001), supporting discriminative validity. No correlations between dual-task performance and DTQ score; do not support construct validity.
Evans et al. (2009) [27]	DTQ	RCT	ABI or ONI, *n* = 10 HC, *n* = 9	44.4 ± 8.5 45.11 ± 9.7	DTQ was used as an outcome measure before and after the intervention. The treatment group: a reduction in the DTQ after the 5 weeks of training (*p* = 0.027); supports responsiveness. The control group: no change in the DTQ.
Gagnon and Belleville (2012) [29]	DAQ	RCT	MCI fixed priority, *n* = 12 MCI variable priority, *n* = 12	67.0 ± 7.8 68.4 ± 6.0	DAQ was used as an outcome measure before and after the intervention. Persons with MCI showed higher scores after the intervention (*p* < 0.05), supporting responsiveness.
Raats et al. (2019) [26]	DTQ	NRCT	MS, *n* = 30 HC, *n* = 30	44.1 ± 10.8 43.9 ± 10.5	DTQ was used as an outcome measure. Persons with MS showed higher scores on DTQ compared to HC (*p* < 0.05), supporting discriminative validity.
Strouwen et al. (2017) [30]	DTSL	RCT	PD, *n* = 121	65.9 ± 9.2	DTSL was used as inclusion criteria. No specific DTSL data was provided.
Tun et al. (1997) [23]	DAQ	CSS	Younger adults with SCI, *n* = 23 Elderly adults with SCI, *n* = 23	39.9 ± 6.8 67.1 ± 3.5	DAQ was used as an outcome measure to compare younger and elderly adults with SCI. No group differences were found, this does not support discriminative validity.
Veldkamp et al. (2019a) [28]	DTSL DTQ	RCT	MS-RR DTT, *n* = 20 MS-RR SMT, *n* = 20	51.4 ± 9.3 53.4 ± 9.2	DTSL was used as a screening method for the inclusion criteria. DTQ was used as an outcome measure before, after, and follow-up the intervention. No significant improvement in DTQ; do not support responsiveness.
Veldkamp et al. (2019b) [12]	DTQ DTSL	NRCT	MS, *n* = 34 HC, *n* = 31	49.3 ± 9.1 48.8 ± 9.1	DTQ was used as an outcome measure to compare persons with MS and HC. Persons with MS showed higher scores on DTQ compared to HC (*p* < 0.001), supporting discriminative validity. DTSL was used as a screening method for the inclusion criteria.
Veldkamp et al. (2021) [24]	DTQ DTSL	NRCT	MS, *n* = 83 HC, *n* = 33	50.8 ± 9.1 48.7 ± 8.9	DTQ was used as an outcome measure to compare persons with MS and HC. Persons with MS showed higher scores on DTQ than HC (*p* < 0.001), supporting discriminative validity. DTSL was used as a screening method for the inclusion criteria.

Abbreviations: PRO = patient-reported outcome; DTQ = Dual Tasking Questionnaire; NRCT = non-randomized controlled trial; MS-RR = multiple sclerosis relapsing-remitting; HC = healthy controls; RCT = randomized controlled trial; ABI = acquired brain injury; ONI = other neurological illness; DAQ = Divided Attention Questionnaire; MCI = Mild cognitive impairment; DTSL = Dual Task Screening List; PD = Parkinson Disease; CSS = cross-sectional study; SCI = spinal cord injury; SMT = Single Mobility Training; DTT = Dual-Task Training.

## Data Availability

Not applicable.
